# Re-epithelialization: advancing epithelium frontier during wound healing

**DOI:** 10.1098/rsif.2013.1038

**Published:** 2014-04-06

**Authors:** M. Ben Amar, M. Wu

**Affiliations:** 1 Laboratoire de Physique Statistique, Ecole Normale Supérieure, UPMC Univ Paris 06, Université Paris Diderot, CNRS, 24 rue Lhomond, 75005 Paris, France; 2 Faculté de médecine, Institut Universitaire de Cancérologie, Université Pierre et Marie Curie-Paris 6, 91 Bd de l'Hôpital, 75013 Paris, France

**Keywords:** chemotaxis, re-epithelialization, wound healing, free-boundary problem, level-set methods

## Abstract

The first function of the skin is to serve as a protective barrier against the environment. Its loss of integrity as a result of injury or illness may lead to a major disability and the first goal of healing is wound closure involving many biological processes for repair and tissue regeneration. *In vivo* wound healing has four phases, one of them being the migration of the healthy epithelium surrounding the wound in the direction of the injury in order to cover it. Here, we present a theoretical model of the re-epithelialization phase driven by chemotaxis for a circular wound. This model takes into account the diffusion of chemoattractants both in the wound and the neighbouring tissue, the uptake of these molecules by the surface receptors of epithelial cells, the migration of the neighbour epithelium, the tension and proliferation at the wound border. Using a simple Darcy's law for cell migration transforms our biological model into a free-boundary problem, which is analysed in the simplified circular geometry leading to explicit solutions for the closure and making stability analysis possible. It turns out that for realistic wound sizes of the order of centimetres and from experimental data, the re-epithelialization is always an unstable process and the perfect circle cannot be observed, a result confirmed by fully nonlinear simulations and in agreement with experimental observations.

## 1. Introduction

Adult skin is made up of three layers: the epidermis and the dermis separated by the basement membrane. When a profound injury occurs which destroys a part of the dermis, it has to be mended rapidly to restore the protective barrier [1,2]. *In vivo* wound healing is a complex repairing process orchestrated by intra- and intercellular pathways. To recover the integrity of the skin, this process is accomplished by four successive but overlapping stages: clotting, inflammation, re-epithelialization and remodelling. Immediately after an injury, a clot composed mainly by fibrin fibres and platelets is formed to plug the wound (stage 1). Subsequently in the next 2–10 days (stage 2) the clot is continuously infiltrated by inflammatory cells which clear up the debris and release chemical factors, such as vascular endothelial growth factor and transforming growth factor-beta. Once the chemical gradient is established, different cells are recruited to fabricate the new tissue (stage 3). On the one hand, neo-vasculature [3] and fibroblasts (depositing collagen) are recruited in the dermis, reforming the clot into a granulation tissue, which nutritiously and physically support the repair of upper layers. On the other hand, keratinocytes migrate and proliferate at the edge of the wound to extend the newly formed epithelial carpet made of several layers of cells in the epidermis. This process is called re-epithelialization and it lasts for two to three weeks. At the end of stage 3, myofibroblasts transformed from fibroblasts contract and try to bring the wound edge together. They disappear by apoptosis in the dermis. The remodelling (stage 4) continues for months or even for years in order to restore the homoeostasis of the normal skin. However, the normal anatomical structure is not truly recovered and a scar is formed from the granulation tissue. While this is true for large wounds for adult humans, those in human embryos [4] may be perfectly closed, the reasons being not fully understood. Interestingly, the scar formation is believed to be an evolutionary sacrifice in order to achieve rapid wound closure [5] and this is indicated by the intense and redundant inflammatory response [6] which mediates the systematic cell behaviours [7] during re-epithelialization through releasing chemical signals. In all cases, the final scar is an outcome of the interplay between cells and the micro-environment [8] through physical and chemical factors during the four stages.

**Figure 1.**
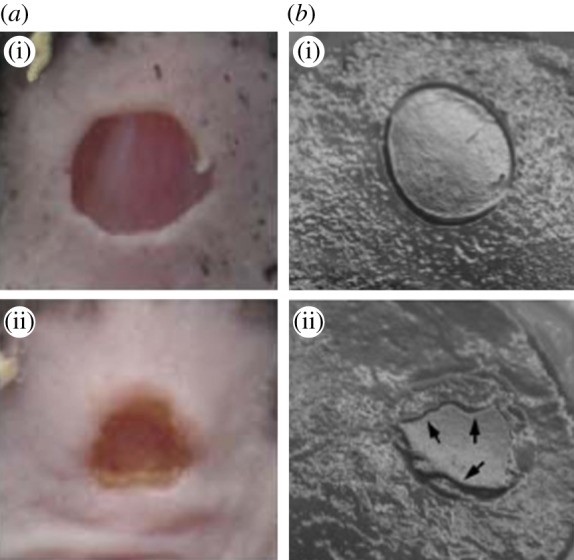
The closing process after a 5 mm diameter punch biopsy on the normal skin of wild-type mice (*a*(i), from [14], copyright with permission) and a 6 mm diameter punch biopsy on an artificial corneal tissue (*b*(i), from [15], copyright with permission). Both present a wavy border as the wound closes. The photographs taken 3 days (for the wild-type mice skin) and 2 days (for the corneal tissue) after the punch are shown in *a*(ii) and *b*(ii), respectively.

Here, we focus on re-epithelialization when keratinocytes, the epidermal predominant cells, detach from the basal membrane, migrate towards the wound margin and proliferate, driven by morphogens in the granulation tissue [9]. Very careful *in vitro* epithelium migration experiments have been performed recently by several groups [10–13]. Realized on a solid substrate, they involve an advancing monolayer motivated by the collective behaviour of cells through migration, generation of forces and reorganization of the cell cytoskeleton at the margin. The findings indicate a non-intuitive manner of cells to achieve robustness during the repair where chemotaxis seems not to be decisive for the global cell migration *in vitro*. Nevertheless, owing to the abundant morphogenic signals released in the wound *in vivo* compared with *in vitro* experiments, chemotaxis dominates the collective behaviour of cells during the wound-healing process. Closer to *in vivo* wound healing (figure 1*a*(i)(ii)), an artificial cornea has been reconstituted [15] (figure 1*b*(i)(ii)) by the introduction of epithelial cells, fibroblasts and growth factors in a three-dimensional Petri dish. Interestingly, all these works [11,12,15] concern the circular geometry rather than the linear cut done in earlier experiments [16,17]. Although well controlled, it is unclear that these systems present the whole complexity of *in vivo* wound healing [14]. Nevertheless, both *in vivo* and *in vitro* experiments present disordered border evolution, which indicates a possible universal irregularity owing to chemotaxis (figure 1).

In this work, we describe the re-epithelialization by considering a circular hole in a tissue, adapting a recent dynamical model of chemotactic migration [18] driven by morphogenetic gradients. Being continuous, the model describes the tissue at a scale larger than the cell size but smaller than the size of the hole. Here, we do not distinguish cell species or the chemoattractant categories. The latter is continuously supplied by an incoming flux from the third dimension normal to the epithelial layer and uptaken by cell receptors. As shown in [18], the thinness of the moving layer transforms the three-dimensional process into a two-dimensional model, where the localized thickness variation at the border contributes to a tension *T*. In addition, we assume that a strong viscous friction exists between the moving layer and the substrate [19–23], which allows writing a Darcy's law for the average velocity field inside the tissue, while cell–cell interactions and mitosis are transformed into interface boundary conditions in the sharp interface limit. We first present the physical model, then the analytical treatment giving an explicit solution for the circular geometry and a study of its stability. Indeed in the case of a linear border, it has been shown that a long wavelength instability occurs at low velocities owing to a Goldstone mode (translational invariance) that surface tension alone cannot succeed to prohibit [18]. Intuitively, one may expect that it disappears for small holes as the surface tension effect accentuates. On the contrary, as for experiments *in vitro*, larger holes may exhibit contour instabilities leading to dynamical behaviours that we aim to predict. To go beyond the stability analysis, the time-dependent free-boundary problem requires numerical methods able to solve equations on evolving domains. We choose to tackle our problem by level-set methods, first developed in the 1980s by Osher & Sethian [24], which are able to track moving interfaces and topological changes automatically and have been successfully applied to problems, such as liquid–gas interactions [25], image processing [26] and tumour growth [27–30]. Owing to the nature of our theoretical problem, we select the methodology developed for tumour growth [27–30]. In the following, we first present the model, introducing the most relevant physical parameters, then the analysis for weak amplitude instability, and finally numerical methods and simulations for the ultimate state of the closure.

## 2. The model

**Figure 2.**
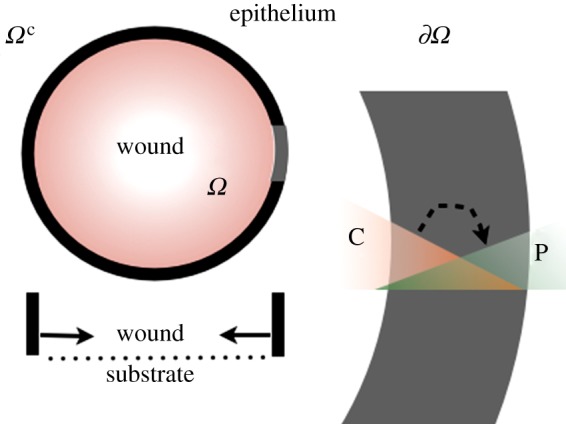
The schematic description: *Ω* is the wound (clot → granulation tissue) where chemoattractants are released and *Ω*^c^ is the moving epithelial continuum. The hydrostatic pressure gradient in *Ω*^c^ is driven by chemotaxis. The proliferation is constrained at the border 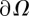 (contour in black and zoomed in by grey).

We consider a circular hole *Ω* in a *two-dimensional* cellular population of density *ρ* immersed in a morphogenetic environment (figure 2). Cells migrate up the morphogen gradient, the chemotactic flux being proportional to the concentration gradient of morphogens 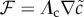, with *Λ*_c_ being a mobility constant. The morphogen repartition 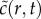 inside and outside the hole *Ω* is alimented through sources coming from below. Mitosis happens only at the border [15,31] and the closure is mostly achieved by the migration. The mass balance for the cell population is then2.1
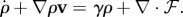

Neglecting volumetric growth (*γ* = 0) in the tissue except at the periphery, the cell density is constant and *ρ* = *ρ*_0_. Equation (2.1) simplifies to 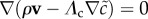 and gives that the normal velocity of the front is directly proportional to the normal concentration gradient. At extremely low velocities, the cell migration satisfies a Darcy's law 
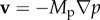
, *M*_p_ being a porosity coefficient equal to the square of the epithelium height divided by a friction coefficient. As shown in [18], this law is deduced in tissues when friction between phases or friction with a substrate balances the hydrostatic part of the elastic stress acting on the cells [18,20]. The wound perturbs the homoeostatic state of the surrounding and a source of morphogens will try to restore an optimal equilibrium value in the aperture *c*_0_. Taking this concentration as unit for the morphogen concentration (giving 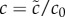), *τ*_c_ the uptake time as time units, *L*_e_ as length unit (with 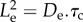, *D*_e_ being the diffusion coefficient inside the epithelium), we get2.2
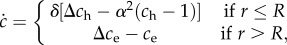
with the index h or e referring to the inner (hole) or outer (epithelium) domain. *δ* = *D*_h_/*D*_e_ represents the ratio between the diffusion coefficient in the hole *D*_h_ and the tissue *D*_e_, it is bigger than 1, and *α* gives the strength of the transverse flux which maintains the morphogen level inside the hole. Owing to the relative slowness of the cell migration, we neglect the time dependence in equation (2.2). Taking *D*_e_/*M*_p_ as pressure unit simplifies Darcy's law into 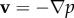, where for simplicity we keep the same notation for *p* and **v**. From mass balance equation (2.1), we get the following Laplace equation coupling the unknown pressure *p* to the chemoattractant concentration *c*:2.3
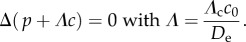
So, we get 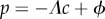, where *ϕ* is a holomorphic function which satisfies Δ*ϕ* = 0. Finally, we must pay special attention to the interface boundary conditions. For equation (2.2), they concern the concentration continuity and the flux discontinuity owing to the morphogen consumption by mitosis at the border 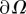 (for demonstration, see [18]):2.4
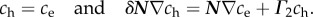
where ***N*** is the outer normal. *Γ*_2_ is the uptake rate constrained at the border discussed below together with the mitosis rates *Γ*_1_ and 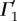 at the border. Capillarity fixes the pressure jump at the interface2.5
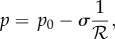
which gives the free-boundary condition considering the geometric effect where 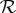 is the local radius of curvature. 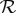 is equal to the radius if the geometry is a circle. However, it considers the local effect at small length scale when the boundary is perturbed. At the same time, the velocity 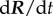 of the interface differs from that of the epithelium **v** by the cell proliferation at the border and we get2.6
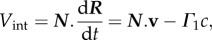
where *Γ*_1_ is the mitosis rate and *σ* the capillary number related to the tension *T* at the interface so 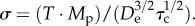 Wound healing *in vivo* is dominated by two-dimensional migration and the morphogens are probably not the nutrients, so *Γ*_2_ vanishes and *Γ*_1_*c* must be replaced by 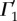 if we also consider mitosis without the quantitative effect of the nutrient concentrations. The capillary number *σ* may involve the activities of actin cable (bundled microfilaments) in the cells. During embryonic wound healing, the leading edge cells can coordinate their actin cables globally which generate an effect on the macroscopic level. However, this coordinated behaviour is lost in adults, so the effect of actin cables may act only locally. This resembles *σ* in our model which is not effective for large wounds. Considering the set of equations (2.2) and (2.3) in conjunction with the set of boundary conditions (2.4), (2.5) and (2.6) shows that chemotatic-driven migration is indeed a free-boundary problem [32,33] involving several parameters to represent the biological complexity and the study of simple cases, for example the circular closure, may help in understanding their corresponding roles. Thus, we present first the analytical results for a hole remaining circular at all times, then its stability.

## 3. The results

### 3.1. Regular circular closure

In the quasi-static approximation, equation (2.2) can be solved analytically and gives3.1
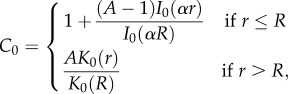


*I*_0_ (resp. *K*_0_) being the modified Bessel function of zero order, regular at *r* = 0 (resp. *r → ∞*). Equation (3.1) takes into account the continuity at the interface, *A* being given by the flux continuity and reads3.2
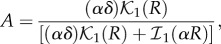
with the following definition for 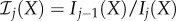 (ratio of two successive Bessel functions) and the equivalent for 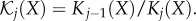 The pressure *P*_0_ inside the epithelium becomes3.3
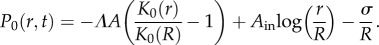
We use the Laplace law to fix the unknown degree of freedom. The holomorphic function *ϕ*, proportional to log(*r*), represents a possible driving force which appears at the interface (i.e. the so-called kenotaxis defined in [13]). Indeed, even in the absence of morphogens, *in vitro* epithelium migration on solid substrate is observed, perhaps owing to the reorganization of the cellular cytoskeleton at the wound margin. Note that the model remains valid if we discard the chemotaxis provided the definition of length and time units is modified. Furthermore, *C*_0_ and *P*_0_ are dependent on time via *R*(*t*). For simplicity, we drop the time dependence of 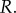 The velocity 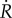 of the closure deduced from equation (3.5) is then3.4
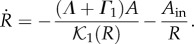


**Figure 3.**
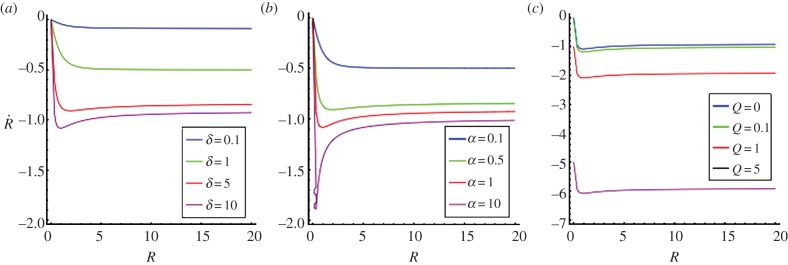
The closing velocity 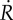 as a function of *R*, varying (*a*) *δ*, (*b*) *α* and (*c*) 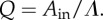 For large *R*, the velocity is constant. The velocity converges to 0 when *R* goes to 0 except that the effect of *A*_in_(>0) is considered (*c*).

The closing velocity is constant for large holes 
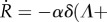
 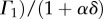 and is exponentially small 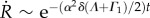 when the radius *R* becomes tiny, if one restricts on chemotatic migration and proliferation. So the radius of a large hole begins with a linear decrease in time but the total closure will take an infinite time for a complete achievement. For small holes, chemotaxis becomes subdominant compared with kenotaxis *A*_in_ at the interface which controls the closure dynamics and the radius satisfies a diffusive law in *t*^1/2^ [12]. Figure 3 shows 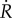 as a function of *R* for different values of *α*, *δ* and *A*_in_/*Λ* and §4 contains a discussion of parameters.

### 3.2. Loss of circularity

**Figure 4.**
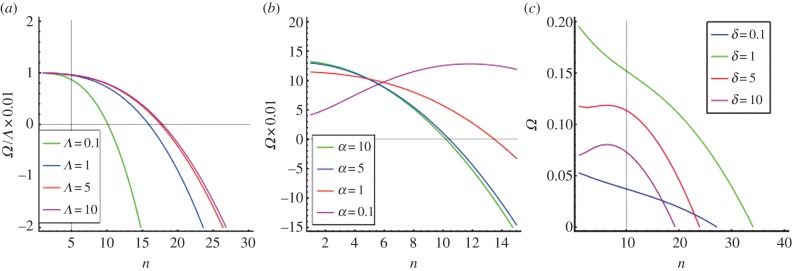
Growth rate *Ω_n_* as a function of wavenumber *n* varying (*a*) the mobility coefficient *Λ*, (*b*) the strength of morphogen *α* and (*c*) the diffusion coefficient ratio *δ* = *D*_h_/*D*_e_ between the wound and the epithelium.

However, equation (3.4) is valid only if the circular contour is maintained. It is why we perform a linear stability analysis for large wounds (*A*_in_ ∼ 0) assuming a small harmonic perturbation for the radius as 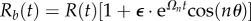 inducing variations on the pressure *P* and concentration field *C* of the same order *ε* as follows:3.5
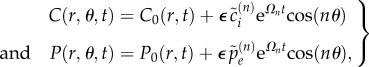
where the subscript *i* indicates either a quantity relative to the hole (h, 0 < *r* < *R*) or to the epidermis (e, *R* < *r* < *∞*). Although all perturbative quantities depend on the selected mode *n*, the linear perturbation analysis treats these modes independently. So we drop the *n* index and calculate the perturbative concentration fields *c_i_*(*r*) from equation (2.3)3.6
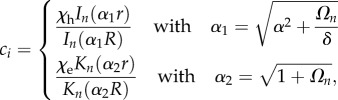
while the perturbed pressure field is given by 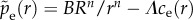 where we take into account the harmonic modes of the holomorphic function *ϕ* with *B* fixed by the Laplace law 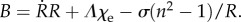 Owing to the weakness of *ε*, our system of equations, once linearized, can be solved analytically and the growth rate of the mode *n* reads3.7
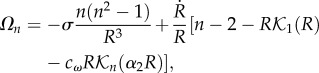
where 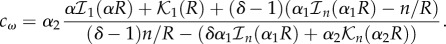
Equation (3.7) represents an implicit relationship for *Ω_n_*, solved by iterative techniques and positive values indicate the modes responsible for the destabilization of the circular border as the migration proceeds. The results are presented for different values of *Λ*, *α* and *δ*, where *Ω_n_* is displayed in figure 4. See §4 for the discussion of parameters. The results indicate an instability leading to a deviation from a circle in short time, for *n* up to a critical mode *n*_c_ (fixed by the capillarity). It is why numerical simulations are necessary to go beyond the linear analysis and fully consider the nonlinearities.

### 3.3. Full dynamics and numerical methods

**Figure 5.**
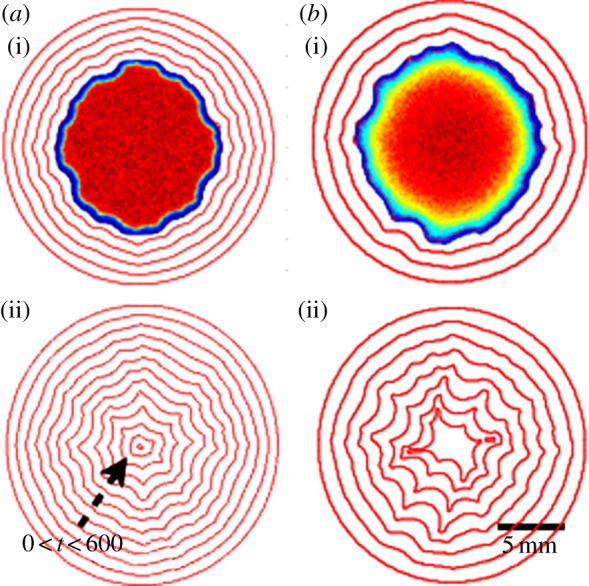
The simulation of wound closing. Both begin with *R* = 90 (9 mm) and *δ* = 2. (*a*)(i) The concentration field and (ii) the overall time evolution with *Λ* = 1 and *α* = 1; (*b*)(i) the concentration field and (ii) the overall time evolution with *Λ* = 10 and *α* = 0.1. Further explanation is given in the text.

**Figure 6.**
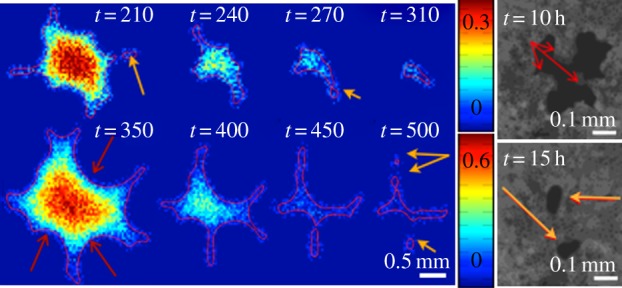
The simulation of the late-stage wound closing with large deformation with *δ* = 2 (top, left) and *δ* = 1 (bottom, left). Right: The MDCK cell monolayer with a wound made by a polydimethylsiloxane cylindrical pillar after 10 (up) and 15 (bottom) hours [12]. The initial diameter of the wound in the experiment is 0.5 mm. The nonlinear border (red arrows) at late stage merges and leads to the pinch-off of wound area (orange arrows) in both the simulation and the experiment.

We discretize *c* in equation (2.2) and pressure *p* in equation (2.3) on a Cartesian mesh in space and implicitly in time, using a nonlinear adaptive Gauss–Seidel iterative method [29,30]. At the domain boundary, we impose both vanishing values of *c* and normal gradient of *p*. Then the noise is added on *C* = *c* + *χ* with 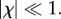 Beginning with a perfect circle with *R* = 90, the wound closing is tracked by the level-set method developed in [27,28] where a scalar function *Φ* with 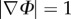 describes the wound (*Φ* < 0), the epithelium (*Φ* > 0) and the interface (*Φ* = 0). The normal and curvature in equation (2.5) are calculated by standard differential geometry: 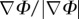 and 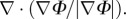 *Φ* is updated by the relation 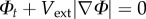, where *V*_ext_ is the constant extension of *V*_int_ on the interface from equation (2.5) following the gradient 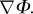 Note that this method simulates a perfect circular closing without noise. According to our results, the wound deviates from a perfect circle soon after the growth as shown in figures 5 and 6 and in agreement with the stability analysis.

## 4. Discussion and conclusion

There are several independent parameters in this model. We can fix some of them with the published experimental data (table 1). Our velocity unit is compatible with the closing velocity of the cornea (3 days for a hole of radius approximately 3 mm [15]) giving *Λ* ∼ 1 for this experiment according to equation (3.2). In figure 4, we vary the parameters *α* and *δ*, which are more difficult to estimate, and also *Λ*. Owing to the large size of wounds in practice, the linear stability analysis gives always an instability and this conclusion is robust to parameter changes. This finding is confirmed by fully nonlinear simulations under the same parameter range (figures 5 and 6). In the simulations, large *Λ* (approx. 10, total time approx. 60) contributes to faster closing compared with small *Λ* (approx. 1, total time approx. 600). A typical example of the closing process with *α* = 1 and *δ* = 2 is shown in figure 5*a*. The advancing interface becomes wavy as the wound heals; however, when the wound becomes small, surface tension re-stabilizes the wound to a potato shape consistent with the linear stability analysis. This surface tension may also pinch wounds off to smaller pieces as shown in figure 5*b*(ii), figure 6 and in [12]. This event requires at first a more irregular closing (red arrows in figure 6), when the concentration of chemoattractant is less homogeneous given inadequate transverse flux (*α* = 0.1). The pinch-off can happen both at the intermediate or the end stage of the closing (yellow arrows), consistent with observations at the late stage of wound healing on a monolayer of MDCK cells (figure 6, right), suggesting the same behaviour in more complex wound-healing processes.

**Table 1.** The parameter table.

physical parameter	value
diffusion coefficient *D*_e_	1 μm^2^ s*^−^*^1^ [34]
uptake time *τ*_c_	2000 s [34]
surface tension *T*	10*^−^*^4^ N m*^−^*^1^ [12]
friction coefficient	10*^−^*^9^ N μm^−3^ [12]
the length unit	50 μm, calculated
the velocity unit	0.025 μm s*^−^*^1^, calculated
the capillary number *σ*	0.1, calculated
the chemoattractant strength *α*	0.1–10, estimated
the diffusion coefficient ratio *δ*	0.1–10, estimated
the cell velocity *Λ*	1–10, estimated

In this work, we have shown theoretically that wound re-epithelialization gives a border instability under clinically realistic parameters. Driven by chemotaxis, our model does not introduce other cell activities which close the wound at the ultimate stage. However, we conjecture that this chemotactic instability at the border of the wound may affect the quality of the final repair. During embryonic wound healing where perfect reconstruction is observed, the wound is closed by a ‘purse string’ which represents a coordination of leading edge cells facilitated by the actin cable [35]. This mechanism is lost in the adult skin, where the wound is closed by the crawling of cells (several layers) on the granulation tissue [36]. Compared with a static substrate *in vitro*, the granulation tissue undergoes contraction at the end of the re-epithelialization by myofibroblasts [37]. Indeed, this purpose is to bring the edge together which resembles the ‘purse string’, but the contraction by those cells needs to be compatible with the synchronous material constitution, which is mechanically and systematically a challenge [7].

At the end, we discuss our model in the context of the skin wound healing *in vivo*. As the wound goes deeply into the dermis which is the case for most wounds, the two layers behave differently during re-epithelialization. The depth of a wound contributes to the thickness in the lower layer where the chemoattractant is released. Considering the re-epithelialization where only several layers of cells become motile above the lower stacks where the chemoattractant is released, the depth of the wound contributes mathematically to the transverse chemoattractant flux strength *α*. Besides *α*, which may involve the depth of the wound, the geometry, the size of the wound as well as the diffusion coefficient ratio *δ* are considered. Comparing with experiments *in vivo*, the human wound-healing process may be different from that in experimental mice owing to different skin biomechanical properties. However, if one intends to consider the problem *in vivo* accounting for the bioelasticity, the challenge does not only come from different components of the two layers, but also the fact that the junction between the dermal and epidermal layer is highly disordered even for the intact skin [38], as shown in our previous work. A regular pattern should be defined in the third dimension which we consider as ‘normal’. Also, as the wound tissue undergoes remodelling during the healing, the mechanical parameters cannot be considered as constants and the relaxation of the tissue probably evolves on a larger time scale than the re-epithelialization. Indeed, these relaxation processes should be also considered in predicting the quality of the final repair. Before that, the irregularity of the wound border driven by chemotaxis should be considered as an ingredient.

## Appendix A. Numerical implementation

The simulation is done on a 200 × 200 Cartesian lattice with a grid size of 10 μm. This free-boundary problem is easy to be implemented with the level-set method owing to its dependence on the curvature in solving the pressure in equation (2.5). The full method is adapted from [28,29] and has an order of accuracy of more than 1.5. While the boundary can be implicitly given by the zero contour of the level-set function *Φ*, the curvature can also be readily calculated. At each time step, the domain from *Ω_t_* to *Ω_t_*_+Δ*t*_ is updated as follows:

— Solve equation (2.2) with the fixed domain *Ω_t_* in the steady state with the free-boundary condition equation (2.4). At the boundary of the computational lattice, the Neumann boundary condition is imposed.
— Update the solution of concentration *C* by *C* = *c* + *χ* pointwisely, where *χ* is randomly generated out of uniform distribution between  and  is used for the presented simulations.
— Solve equation (2.3) with known *C* under the interfacial boundary condition from the first part of equation (2.5), where the local curvature  is calculated from the level-set function by  The Neumann boundary condition is imposed at the boundary of the computational lattice.
— Calculate the velocity *V* of the moving boundary from the second part of equation (2.4), where  is calculated from the level-set function by
— Find the appropriate Δ*t* given by the CFL condition by  and update the domain *Ω_t_* to *Ω_t_*_+__Δ*t*_.
— Start with the newly updated domain *Ω_t_*_+Δ*t*_ and repeat the last five steps.
